# The Reciprocal Relationships Between Escalation, Anger, and Confidence in Investment Decisions Over Time

**DOI:** 10.3389/fpsyg.2018.01136

**Published:** 2018-07-05

**Authors:** Alexander T. Jackson, Satoris S. Howes, Edgar E. Kausel, Michael E. Young, Megan E. Loftis

**Affiliations:** ^1^Department of Psychology, Middle Tennessee State University, Murfreesboro, TN, United States; ^2^Department of Management, Oregon State University, Bend, OR, United States; ^3^School of Management, Pontifical Catholic University of Chile, Santiago, Chile; ^4^Department of Psychological Sciences, Kansas State University, Manhattan, KS, United States

**Keywords:** escalation of commitment, sequential decision making, confidence, anger, judgments

## Abstract

Research on escalation of commitment has predominantly been studied in the context of a single decision without consideration for the psychological consequences of escalating. This study sought to examine (a) the extent to which people escalate their commitment to a failing course of action in a sequential decision-making task, (b) confidence and anger as psychological consequences of escalation of commitment, and (c) the reciprocal relationship between escalation of commitment and confidence and anger. Participants were 110 undergraduate students who completed a series of investment decisions regarding a failing endeavor. Results revealed that although a high proportion of individuals escalate through all decisions, the extent to which they escalated decreased with each decision as they were less willing to invest money in the project. Furthermore, as participants escalated, confidence in one’s decision decreased and anger increased. Lastly, the analyses revealed that the relationship between escalation and confidence is reciprocal. Escalation was negatively associated with confidence, and confidence predicted escalation in the subsequent decision. These results highlight the importance of considering both the determinants and psychological consequences of escalation of commitment.

## Introduction

“You gotta know when to hold ‘em, know when to fold ‘em, know when to walk away, know when to run.”

-Kenny Rogers, The Gambler

The above lyric epitomizes the psychological bias of escalation of commitment. Escalation of commitment refers to the tendency to invest additional resources into an ongoing effort, when doing so is no longer rational (Staw, 1976; Sleesman et al., 2012). Continuing to invest funds into a failing project can result in large financial and productivity losses (e.g., Arkes and Blumer, 1985; Ross and Staw, 1993; Schmidt and Calantone, 2002; Drummond, 2014). Furthermore, research has demonstrated that raters will positively bias their assessments of those employees they were responsible for hiring, thereby demonstrating that escalation of commitment is an issue within personnel selection and assessment decision contexts (Schoorman, 1988). Consequently, considerable attention has been directed toward understanding the factors that influence the likelihood of escalating commitment to a failing course of action. For example, Sleesman et al. (2012) reviewed over 160 studies and found strong evidence that previous resource expenditures, such as money and time, are associated with higher levels of escalation.

Despite the abundance of research on escalation of commitment, the majority has examined a single decision to escalate, despite organizational decision makers often facing repeated decisions about underperforming projects or personnel. Some research has shown a clear trend that individuals initially escalate their commitment to a failing course of action, especially if they were responsible for the initial decision (Staw, 1976). However, the results regarding what happens *after* the initial escalation are mixed. Staw and Fox (1977) found that the relationship between time and escalation resembled a U-shaped function. Immediately following the initial investment decision, participants invested additional funds into a failing venture, thereby exhibiting a strong escalation bias. After receiving additional negative feedback about the venture, participants tended to de-escalate by investing less money in the next decision. When presented with a third decision, however, participants invested significantly more money than the second decision. In contrast, McCain (1986) demonstrated that over the course of 10 financial decisions, individuals tended to escalate their commitment with the first decision after failure, and de-escalate with subsequent decisions.

In addition, the research examining escalation of commitment has predominantly focused on the antecedents of escalating, lacking an evaluation of what happens after escalation has occurred (Schoorman and Holahan, 1996; Sleesman et al., 2012). Specifically, most studies have focused primarily on understanding the influence of project characteristics and psychological factors on the decision to escalate (Sleesman et al., 2012). Little attention, however, has been paid to other aspects of escalation situations, such as the psychological consequences of escalating. These consequences are important, as consequences of escalating might impact future decisions. For instance, while recent research has shown that (over)confidence indeed positively predicts escalation behavior (e.g., Ronay et al., 2017) what is currently unknown is how escalation behavior impacts subsequent confidence. We attempt to fill this gap in the literature by examining two psychological consequences: confidence and anger.

Another area of research that warrants additional focus is whether participants would choose to continue with or abandon the failing project and the repercussions of this decision to abandon. Staw and Fox (1977) allowed their participants to invest $0 in a failing project, whereas McCain (1986) permitted their participants to choose to quit the simulation after the third decision. The current study seeks to extend the findings of Staw and Fox (1977) and McCain (1986) by allowing participants to abandon the project after the first decision and throughout the subsequent decisions. Though Staw and Fox (1977) allowed participants to invest $0 from the beginning, the psychological difference between investing $0 and abandoning the project could be impactful. For example, investing $0 in the project likely leads a participant to believe that the project will continue, just without the additional funds requested. Participants may even feel as though they could invest additional funds into the project later, if the project started to become more promising. In contrast, abandoning the project means that the project will end and the participant is completely giving up hope that the project may be successful. Proceeding in this manner allows us to not only evaluate participant decisions, but also judgments. According to Bonaccio and Dalal (2006), a decision is a choice – such as a participant’s choice to invest additional funds or abandon the project. In contrast, a judgment is a quantitative value – such as the amount of funds actually invested, which may reflect their confidence in the decision to invest.

In summary, there are three purposes of the present study. First, we aim to examine how escalation of commitment unfolds over a series of decisions. Second, we seek to evaluate the degree to which participants escalate to a failing venture by using both their decision to continue funding and their judgment regarding how much to invest. Lastly, whereas previous studies have focused on the determinants of escalation, we evaluate the psychological consequences of anger and confidence, as well as their reciprocal nature, on future escalations in a sequential decision-making context.

### Escalation of Commitment

By definition, escalation of commitment – the decision to invest resources toward an endeavor that one knows is failing – is irrational. When faced with the decision to continue with a course of action or abandon a failing endeavor, the rational choice would be to ignore the time, energy, and resources already invested (i.e., the sunk costs) and abandon the project. Nevertheless, people *do* heavily consider sunk costs and often make the irrational choice of escalating their commitment to failing endeavors (Conlon and Garland, 1993).

The act of escalating itself is not necessarily irrational. Sunk cost effects do not only depend on escalation behavior; they also require an examination of the rationale for such behavior. There may be multiple possible forces promoting escalation behavior, such as overconfidence, the presence of sunk costs, the social and reputational damage of admitting failure, organizational or political barriers, and the need for self-justification (Drummond, 2014). At the same time, there may be multiple possible factors placing pressure on the decision maker to abandon the endeavor, such as one’s own loss aversion, being perceived as wastefully expending resources that could be spent on other opportunities, and the political pressures associated with publicly stated limits or stopping points (Drummond, 2014). Therefore, one must consider the reasons for escalating to determine whether sunk cost effects are occurring.^1^

One of the most promising explanations for initial escalation behavior involves self-justification theory (Brockner, 1992). Opting to terminate a failing project or dismiss an ineffective employee would create cognitive dissonance for individuals, as their decision would be counter to their initial belief that the project or employee would be a success. As such, individuals who were responsible for the initial decision attempt to justify their actions in order to reduce the cognitive dissonance they are experiencing (Sleesman et al., 2012). By rationalizing their behavior and electing to continue with the failing project/employee (hoping things will ultimately turn around and initial beliefs will be justified), individuals are able to protect their ego (Zhang and Baumeister, 2006) and reduce any further cognitive dissonance. For instance, when faced with an escalation decision, concerns about one’s own reputation may be heightened (Zhang and Baumeister, 2006; Sleesman et al., 2012). The heightened reputational concerns activate the need to justify one’s decisions. If a previously decided course of action is now failing, the decision maker may feel that his or her reputation is going to be harmed because he or she is not succeeding. This may then lead to the need to justify one’s previous actions by staying the course with the hopes of eventual success. Indeed, ego threat (i.e., reputational threat) has been shown to be one of the strongest predictors of escalation (Sleesman et al., 2012).

In support of the self-justification theory, Arkes and Blumer (1985) demonstrated that the money already invested in a particular venture leads people to experience pressure to justify their actions to themselves. In an effort to avoid appearing wasteful, individuals opt to continue a course of action with the hope that the venture will ultimately be successful. In a similar Wong and Kwong (2007) found that when anticipated regret for abandoning a project early was high, individuals were more likely to escalate their commitment. As such, anticipated regret appears to serve as self-justification mechanism for escalation. These are only two of many determinants possible mechanisms for self-justifying escalation behavior. For instance, in their meta-analysis, Sleesman et al. (2012) found support for a number of psychological determinants of escalation of commitment: sunk costs, time investment, personal experience or expertise, self-efficacy/confidence, responsibility for the initial decision, ego threat, and anticipated regret. Each of these determinants relies on self-justification theory to explain why people escalate. Accordingly, Sleesman et al. (2012) argued that self-justification theory has merit as a central theory explaining *why* individuals choose to escalate their commitment toward failing endeavors.

### Escalation in Sequential Decisions

One of the key characteristics of decision making in applied settings is that many dilemmas, including escalation decisions, are not resolved after a single decision. Even the dilemma of whether to continue with or abandon a failing endeavor may have multiple decision points. For example, after making an initial investment decision, an employee may receive negative feedback that triggers self-justification processes and the decision to invest additional resources. After those resources are used, the project may still be failing, and additional decisions may be required to continue the project. Initial decisions, the associated outcomes of those initial decisions, subsequent decisions, and outcomes all unfold over time. Thus, it is important to examine the extent to which people are willing to repeatedly escalate.

As noted previously, self-justification theory argues that individuals feel compelled to justify their previous behavior, which in turn leads to escalation. In the case of a sequential decision-making situation, individuals may repeatedly receive information that their previous decisions were poor, continuously reviving the cognitive dissonance experienced (Draycott and Dabbs, 1998). Accordingly, individuals would be expected to engage in self-justification processes and continue escalating to alleviate the renewed dissonance experienced at each decision point. However, over time, it may become more difficult to rationalize one’s actions and reduce the cognitive dissonance. Instead, the only feasible option becomes reducing the investment in a project, and ultimately discontinuing one’s commitment despite sunk costs.

In addition, cognitive dissonance may lead individuals to experience psychological discomfort (Festinger, 1957; Elliot and Devine, 1994), negative affect (Harmon-Jones, 2000), and physiological arousal (Elkin and Leippe, 1986). However, even when individuals engage in strategies to alleviate these negative sequelae, they may not experience a decrease in arousal (Elkin and Leippe, 1986). Therefore, although individuals engage in self-justification processes, it is likely that they do not experience a decrease in psychological discomfort. As such, we expect the discomfort experienced from repeatedly learning that one’s decisions are not leading to success will result in increased anger at the decision, and reduced confidence in one’s ability to make good decisions. Support for the above notion comes from the feedback literature. Both Tata (2002) and Belschak and Den Hartog (2009) documented the effect of performance feedback from managers on employees’ affect and found that when performance feedback was negative, employees tended to experience more anger. In line with this, we predict that as decisions unfold over multiple decision points and negative feedback continues, individuals are less likely to escalate their commitment (both in terms of commitment to the project and monetary investment toward the project) and are likely to become increasingly angry and decreasingly confident in their decisions. This leads to the following hypotheses:

*Hypothesis 1:* Escalation of commitment toward a failing course of action will decrease over time (multiple decision points).
*Hypothesis 2:* The degree to which individuals escalate (invest money) toward a failing course of action will decrease over time (multiple decision points).
*Hypothesis 3:* Continued escalation over multiple decisions will lead to increased anger.
*Hypothesis 4:* Continued escalation over multiple decisions will lead to decreased confidence.

**FIGURE 1:**
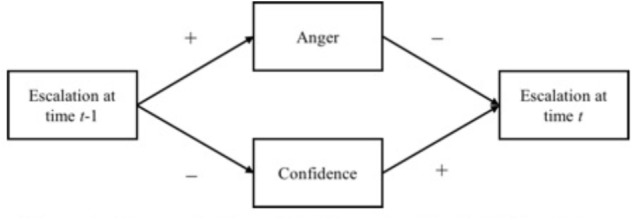
Conceptual model of hypothesized relationships.

Further, these relationships may be reciprocal in nature. Supporting this idea, Van Overwalle and Jordens (2002) argued that individuals rely on information about their affective experiences when making judgments. According to affective events theory, work environment features influence work events, work events lead to affective reactions, and affective reactions ultimately lead to affect driven behaviors (Weiss and Cropanzano, 1996). Affective events theory can be used to explain the determinants of escalation by examining the work environment features (i.e., features of the decision), the psychological outcomes of work events (i.e., electing to escalate), and future behaviors. Specifically, when an individual receives negative feedback about a course of action and elects to continue with the course of action, he or she would likely experience the hypothesized changes in confidence and anger. The hypothesized decrease in confidence would then lead an individual to actually abandon the failing course of action sooner. In other words, because an individual becomes less confident in their decision-making capabilities as a result of escalating, he or she would be less likely to continue escalating, resulting in a downward spiral of confidence and escalation. Similarly, the hypothesized increase in anger resulting from escalating likely leads an individual to abandon the endeavor sooner, rather than later (**Figure 1** displays this conceptual model). Indeed, Strough et al. (2016) found that negative affect is positively related to willingness to cancel a failing plan. This leads to the following hypotheses:

*Hypothesis 5:* The lagged effect of anger negatively predicts escalation in the next decision.
*Hypothesis 6:* The lagged effect of confidence positively predicts escalation in the next decision.

## Materials and Methods

### Participants

Participants were 110 (32 males, 78 females) undergraduate students recruited from a large Midwestern university. The sample was primarily White, non-Hispanic (84%), with a mean age of 20 (*SD*= 3). Approximately 52% of the sample was employed at least part-time.

### Procedures

The study was conducted individually in a laboratory setting. The decision task was based on the “blank radar plane” case originally presented by Arkes and Blumer (1985) and widely used to study escalation of commitment (Conlon and Garland, 1993; Moon, 2001a,b; Wong et al., 2006). For this task, participants are presented with a vignette in which they are asked to assume the role of the Vice President of Operations for a mid-sized high-technology manufacturing firm. Because we were interested in examining how escalation of commitment occurs over time, we created additional decision vignettes that followed the first decision (each of the decision vignettes are presented in **Appendix A**). All information was presented in printed text format.

Participants were presented with the first scenario and asked to make the initial investment decision. Specifically, they were asked, “Between 5 million dollars and 10 million dollars, how much money would you like to invest in the project?” For each subsequent decision, participants were presented with additional information indicating that the project was still not complete and needed additional funds (see **Appendix A**). Participants were then asked whether they wanted to authorize more funds to continue the project or abandon the project. The specific instructions stated, “The decision you face now is to either abandon the project or authorize more funding to continue this radar-scrambling project.” Thus, the escalation variable was dichotomous (continue the project vs. abandon the project). Additionally, if participants chose to authorize more funding, they were asked how much money they wished to authorize, based on the information from the vignette. The amount of money participants could choose to authorize differed throughout the five exercises and was based on the current state of the project in order to increase the fidelity of the task (see **Appendix A**). See **Table 1** for the differing funding available at each decision point. If individuals chose to pursue the failing project at all decision points, they would be asked to make a total of five decisions regarding the funds to be authorized and four decisions regarding whether to continue with the project (the escalation vignettes are available upon request from the authors). Immediately following each decision point, participants were asked to indicate how confident they were in their decision and to complete a brief measure of emotions. Performance in the task was not incentivized. The study ended after a participant continued through all five decisions or abandoned the project.

**Table 1:** Funding available at each decision point.

Decision 1	$5 million – $10 million
Decision 2	$3 million – $6 million
Decision 3	$2 million – $5 million
Decision 4	$4 million – $7 million
Decision 5	$1 million – $4 million

### Measures

#### Confidence

Confidence was measured using two items adapted from Greer and Stephens (2001) confidence scale. After each decision point, participants indicated how confident they felt in their decision using a 1 (*Low Confidence*) to 7 (*High Confidence*) scale. An example item stated, “How confident are you in your resource investment decision?” Responses to the two items were averaged to create a composite confidence score for each decision point. The average coefficient alpha for the confidence scale across the five decision points was 0.92.

#### Anger

To assess how anger changes over time, we used the four anger-related items (i.e., Angry, Furious, Mad, and Frustrated) from the State Trait Anger Expression Inventory (Spielberger et al., 1988). After each decision, participants were asked, “When thinking about the events that led to you having to make this decision, how are you feeling right now?” Participants responded using a 1 (*Not at all like me*) to 5 (*Very much like me*) scale. In order to minimize any priming effects for the subsequent decision, the anger items were interspersed with an equal number of positively valenced items. These positive items were not examined because they were used to minimize demand characteristics. The average coefficient alpha for the state anger scale across the five decision points was 0.83.

#### Additional Measures Not Included in the Analyses

This study was conducted as part of a larger study examining escalation of commitment in general. As a part of this larger study, data on additional variables were also collected during the data collection. These variables include a variety of personality traits, including: generalized self-efficacy, grit, regulatory focus, goal orientation, narcissism, psychopathy, Machiavellianism, trait affectivity, trait regret, guilt proneness, and the big five. Additionally, participant’s state level of pride was measured. However, the focus of this study was exclusively on the state changes in confidence and anger as people escalate. As such, personality characteristics and pride were excluded from the analyses.

## Results

**FIGURE 2:**
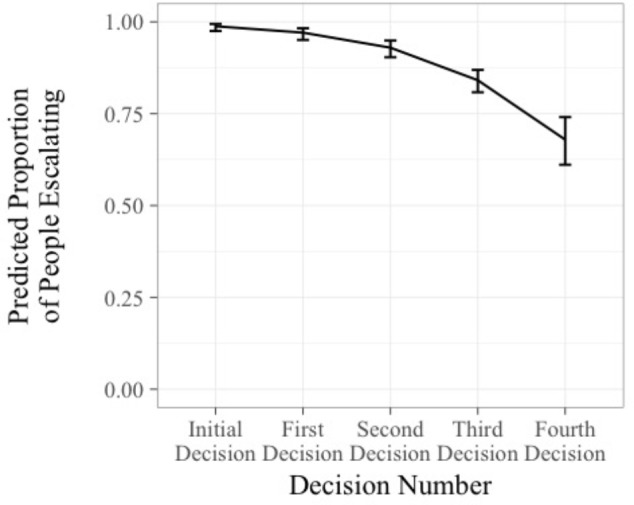
Predicted probability of escalating commitment over time. Error bars represent ±1 standard error.

Correlations between all variables of interest are displayed in **Table 2**. First, we examined how long individuals were willing to continue with a failing course of action. A repeated measures logistic regression was conducted using the generalized linear mixed-effects modeling package in *R* (Bates et al., 2014). A logistic regression was selected because escalation was measured as a dichotomous variable (authorize funds vs. abandon project). Because the current analysis assessed escalation over time, decision number (the effect of time) was entered as a continuous fixed effect, participant was entered as a random intercept effect^2^, and escalation was entered as the criterion. The results of the analysis revealed that decision number was significantly negatively associated with escalation of commitment, *B*= -0.92, z = -4.02, *p* < 0.01, 95% CI [-1.36, -0.47]. In other words, as the project progressed and negative feedback continued, participants became less willing to continue with the project, supporting Hypothesis 1. As shown in **Figure 2**, the predicted proportion of individuals escalating their commitment after the initial investment decision is approximately 0.98, yet over time, this proportion significantly decreased to 0.68 at the fourth decision after the initial investment decision.

**Table 2:** Correlations among variables.

Variable	1	2	3	4
(1) Decision number	–	–	–	–
(2) Escalation	**–0.27**	–	–	–
(3) Proportion invested	**–0.33**	**0.11**	–	–
(4) Confidence	**–0.17**	0.07	**0.18**	–
(5) Anger	**0.29**	**–0.12**	**–**0.05	**–0.33**

*Bolded values are significant at p < 0.05.*

Next, we examined how the amount of money individuals are willing to invest changes over time. Participants were given a range of funds to invest at each decision point. Because different ranges of available funds were used at each decision point based on the scenario, the amount of money each person invested was transformed into the proportion of money invested out of the total amount available at that decision point. For individuals who selected the maximum or minimum values of the range of funds available, their proportions were 1.00 and 0.00, respectively. Data that are scored from 0.00 to 1.00, such as proportions, often accumulate heavier at the 0.00 and 1.00 values than the values of a normal distribution, necessitating a logit transformation to extend the tails of the distribution (Cohen et al., 2003). With a logit transformation, data equaling 0.00 and 1.00 become negative and positive infinity, respectively, in the transformed space, which necessitates adding 0.05 to any 0.00 values and subtracting 0.05 from any 1.00 values. Thus, for each of the proportions that were 1.00 or 0.00, 0.05 was subtracted or added, such that adjusted scores ranged from 0.05 to 0.95. These adjusted scores remained the highest and lowest values on the scale. The proportion of funds invested variable was then logit transformed.

**FIGURE 3:**
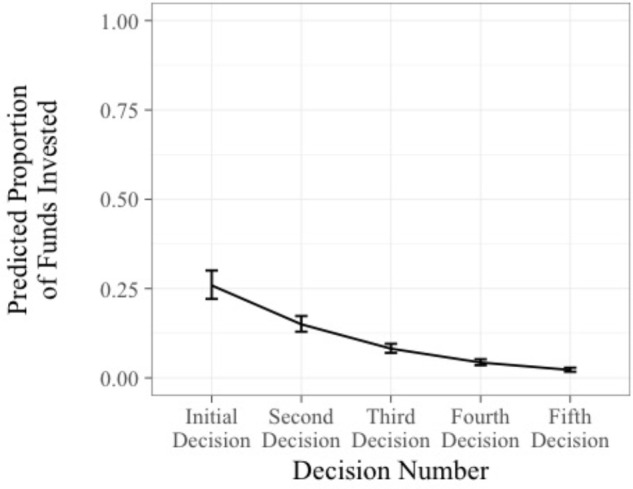
Predicted proportion of funds invested over time. Error bars represent ±1 standard error.

A linear mixed effects regression was then conducted using the linear mixed-effects modeling package in *R* (Bates et al., 2014). As in the first analysis, because we are examining changes over time, decision number (the effect of time) was entered as a fixed effect, participant was entered as a random intercept effect, and the proportion of funds invested was entered as the criterion. We also evaluated models that included decision number (the time slope) as a random slope effect. However, because the model excluding decision number as a random effect resulted in a better fitting model (i.e., lower BIC), we report and interpret the results of model with decision number entered as only a fixed effect. Results revealed that decision number was negatively associated with the proportion of funds people were willing to invest, *B* = -0.68, *t*(428) = -8.44, *p* < 0.05, 95% CI [-0.84, -0.52]. Thus, as the course of action continues to fail, participants became increasingly less willing to invest money over time, supporting Hypothesis 2. As shown in **Table 3** and **Figure 3**, the predicted proportion of available funds initially invested was 0.26 ($6.3 Million when choosing from the $5 to $10 million range). However, as participants continued to escalate their commitment over time, the predicted proportion of funds approached the minimum of the offered range. In other words, at the initial investment decision, participants were willing to invest more than the first quartile of the available funds. However, as they continued to escalate their commitment, willingness to invest dropped substantially. At the final decision, individuals invested near the minimum of the available funds.

**Table 3:** Proportion of funds invested over time.

	Available funds	Relative proportion invested	Average amount invested
Decision 1	$5 million – $10 million	0.26	$6.3 million
Decision 2	$3 million – $6 million	0.15	$3.45 million
Decision 3	$2 million – $5 million	0.08	$2.24 million
Decision 4	$4 million – $7 million	0.04	$4.12 million
Decision 5	$1 million – $4 million	0.02	$1.06 million

Hypotheses 3 and 4 stated that individuals would experience increased anger and decreased confidence, respectively, as they continue to escalate their commitment toward the failing project. Separate analyses were conducted with anger and confidence as the criterion in each analysis. According to Bonaccio and Dalal (2006), a decision is a choice – such as a participant’s choice to invest additional funds or abandon the project. In contrast, a judgment is a quantitative value – such as the amount of funds actually invested, which may reflect their confidence in the decision to invest. We operationalized escalation both as a decision (continue funding) and a judgment (proportion invested). Therefore, we conducted two separate linear mixed effects regressions. In both analyses, decision number (the effect of time) was entered as a fixed effect and a random slope effect, and participant was entered as a random intercept effect. The operationalization of escalation was entered as a fixed effect. We also examined models with the operationalization of escalation entered as a random slope effect. However, in all of the models examined, the best fitting model (i.e., lowest AIC and BIC) did not include escalation as a random slope effect. Therefore, we report the results of the models with the operationalization of escalation entered as a fixed effect only.

**FIGURE 4:**
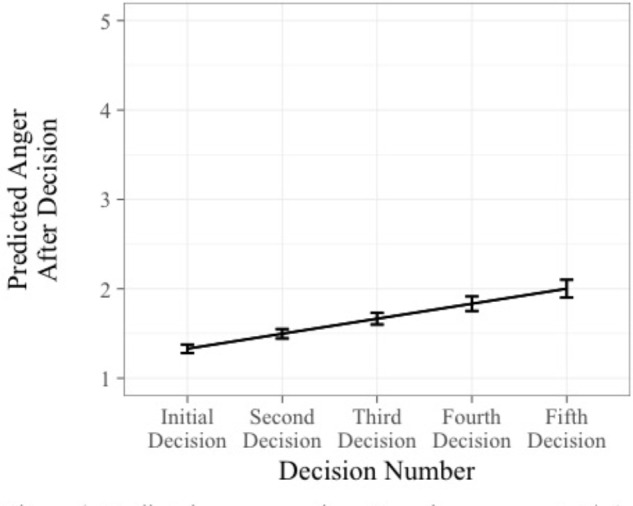
Predicted anger over time. Error bars represent ±1 standard error.

The first set of analyses examined anger as the criterion. The first regression operationalized escalation as a decision and revealed that the decision to escalate was not a significant predictor of anger, *B* = -0.11, *t*(481) = -1.19, *p* > 0.05, 95% CI [-0.28, 0.07]. However, decision number was positively associated with anger, *B* = 0.16, *t*(481) = 6.50, *p* < 0.05, 95% CI [0.11, 0.20]. Thus, with each decision people became increasingly angry (see **Figure 4**). The second regression operationalized escalation as a judgment by using the proportion of funds invested variable. This second regression revealed a similar pattern. The proportion of funds invested did not significantly predict anger, *B* = -0.02, *t*(428) = -1.43, *p* > 0.05, 95% CI [-0.04, 0.01], but decision number was positively associated with anger. Therefore, Hypothesis 3 was not supported.

**FIGURE 5:**
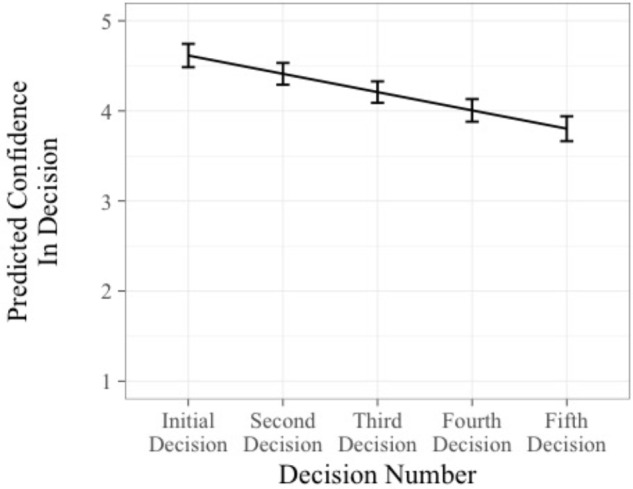
Predicted confidence over time. Error bars represent ±1 standard error.

The second set of analyses examined confidence as the criterion. The first regression operationalized escalation as a decision and showed that the decision to escalate did not significantly predict confidence in the decision, *B* = -0.23, *t*(476) = 1.48, *p* > 0.05, 95% CI [-0.54, 0.08]. However, decision number significantly predicted confidence, *B* = -0.23, *t*(476) = -5.90, *p* < 0.05, 95% CI [-0.31, -0.16]. This suggests that as individuals continue with a failing course of action, they become decreasingly confident with each decision that they are making (see **Figure 5**). The second regression, operationalizing escalation as a judgment, revealed that the proportion of funds invested *did* significantly predict confidence, *B* = 0.06, *t*(423) = 2.97, *p* < 0.05, 95% CI [0.02, 0.10]. Furthermore, decision number was negatively associated with confidence, *B* = -0.19, *t*(423) = 4.67, *p* < 0.05, 95% CI [-0.27, -0.11]. Thus, as people invested more money in each decision, they felt more confident in the decision. However, on average confidence still decreased with each decision. Thus, Hypothesis 4 was partially supported.

### Lagged Effects of Anger and Confidence

In order to test Hypotheses 5 and 6, the lagged effects of anger and confidence on escalation behavior were examined. Specifically, we examined the effect of anger and confidence at time *t-1* on the decision to escalate at time *t* in separate analyses. The analyses were conducted using the linear mixed effects modeling and generalized linear mixed effects modeling packages in *R*. Additionally, like in the analyses testing Hypotheses 3 and 4, separate analyses were conducted for the separate operationalizations of escalation.

Hypothesis 5 stated that the lagged effect of anger would be negatively associated with escalation in the next decision. To test this, the operationalization of escalation at time *t* was predicted by anger at time *t–1* and decision number at time *t*. Specifically, anger at time *t–*1 and decision number were entered as fixed effects, and participant was entered as a random intercept effect. The lagged effect of anger at time *t-1* did not significantly predict whether one chose to escalate at time *t, B <*0.01, *z*= 0.02, *p*> 0.05, 95% CI [-0.46, 0.47]. However, decision number significantly predicted the choice to escalate at time *t, B* = -0.65, *z*= -2.74, *p*< 0.01, 95% CI [-1.11, -0.18]. A similar pattern emerged when examining escalation as the amount of funds invested. The lagged effect of anger at time *t*-*1* did not significantly predict the funds one invested at time *t, B* = 0.39, *t*(372) = -1.80, *p*> 0.05, 95% CI [-0.04, 0.81]. However, decision number significantly predicted the funds one invested at time *t, B* = -0.32, *t*(372) = -2.74, *p*< 0.05, 95% CI [-0.55, -0.09]. It appears that although people get increasingly angry with each decision, the increased anger does not influence the choice to escalate or the funds one chooses to invest in subsequent decisions. Therefore, Hypothesis 5 was not supported.

Hypothesis 6 stated that the lagged effect of confidence would be positively related to escalation in the next decision. To test this, the decision to escalate at time *t* was predicted by confidence at time *t*-*1* and decision number at time *t*. Specifically, confidence at time *t*-*1* and decision number were entered as fixed effects, and participant was entered as a random intercept effect. The lagged effect of confidence at time *t*-*1 did* significantly predict whether one chose to escalate at time *t, B* = 0.27, *z*= 2.00, *p*< 0.05, 95% CI [0.01, 0.54]. Additionally, decision number significantly predicted the choice to escalate at time *t, B* = -0.62, *z*= -2.61, *p*< 0.01, 95% CI [-1.08, -0.15]. In contrast, the lagged effect of confidence at time *t*-*1 did not* significantly predict the funds invested at time *t, B* = 0.09, *t*(316) = 0.81, *p*> 0.05, 95% CI [-0.13, 0.32]. Although people become decreasingly confident with each decision, the decreased confidence does not influence the choice to escalate in the next decision. However, individuals who were more confident after each decision invested slightly more of the available funds in the next decision. Therefore, Hypothesis 6 was partially supported.

## Discussion

The purpose of this study was threefold. First, we sought to examine the extent to which people continue escalating their commitment in a sequential decision-making situation. The results of this study revealed that when faced with sequential escalation decisions, fewer and fewer individuals escalate their commitment over time. However, as can be seen in **Figure 2**, a substantial 68% of participants never abandoned the project and escalated through all five decisions. Thus, it seems that when faced with an escalation of commitment dilemma, individuals are highly likely to continue pursuing the failing course of action over time. Furthermore, the results demonstrated that the degree to which individuals tended to escalate decreased over time, as they invested decreasing proportions of the available funds with each decision. These findings demonstrate that while individuals do continue with a failing course of action, they become less willing to invest money in the endeavor with each decision. This may indicate that people feel a decreasing amount of confidence in their ability to turn the project around. Alternatively, the adaptive learning strategies model proposed by Wong and Kwong (2017) may be used to explain our findings. Specifically, they argue that the probability that one will escalate his or her commitment is a function of learning at the strategy level, not the individual decision level. If a specific strategy (e.g., escalation) is reinforced, the individual should continue that strategy. However, if the strategy is not reinforced, the individual should change strategies. In our present study, the escalation was not reinforced. Indeed, the escalation strategy in our study was punished with repeated negative feedback. As such, we see the increasing degree of abandonment with later decisions, even though 68% of participants never abandoned the project.

Second, we sought to examine two psychological consequences (anger and confidence) of escalating one’s commitment to a failing course of action. In the current study, we examined anger and confidence as psychological outcomes and operationalized escalation as a decision (continue to escalate) or a judgment (proportion of funds invested). According to self-justification theory, electing to abandon or reduce the amount of funding to a failing project would create cognitive dissonance for individuals because this would contradict their initial belief that the project would succeed. This cognitive dissonance leads to psychological discomfort, such as anger (Harmon-Jones, 2000). As such, we argued that individuals may actually experience an increase in anger and a decrease in confidence with each subsequent decision or judgment as this revitalizes the initial cognitive dissonance and psychological discomfort. While our results showed that neither the decision to escalate nor the judgment regarding the funds to invest significantly predicted anger, individuals did become increasingly angry with each escalation decision. This may be the result of repeatedly receiving negative feedback because their decisions kept resulting in negative outcomes and the project was not succeeding. Indeed, Belschak and Den Hartog (2009) found that receiving negative feedback led to increased negative affect.

Regarding confidence, we found that the decision to escalate did not significantly predict confidence. However, the judgment regarding the funds to invest significantly predicted confidence, such that as people invested a higher proportion of the funds they felt more confident. Interestingly, as people continued with each decision, they became decreasingly confident. Again, this is likely the result of repeatedly receiving negative feedback. As previously noted, people decreased the proportion of available funds they invested over time. We argue that the decrease in investment with each decision may be a reflection of the decrease in confidence.

The third and final purpose of this study was to explore the lagged effects of confidence and anger on one’s decision to escalate in future decisions and one’s judgment of the amount of money to invest in future decisions. We found that the lagged effect of anger did not influence one’s future decision to escalate or the proportion of funds invested in the next decision. This suggests that while people continue to get angry, perhaps as a result of the negative feedback or lack of success, the increasing anger may simply be reactionary and does not influence their subsequent decisions. In other words, people get angry about their past decisions but do not let it affect their future decisions. Our results are similar to the findings of Tsai and Young (2010). Specifically, they found that although individuals induced to feel angry were significantly more likely to escalate their commitment, they were not significantly more likely to escalate than individuals induced with a neutral emotional prime.

Although our hypotheses regarding anger were not supported, they are in line with the Appraisal Tendency Framework (ATF). According to the ATF, emotions, such as anger, carry motivational properties that influence judgments and decisions (Han et al., 2007). Furthermore, these motivational properties influence the contents of a person’s thoughts, such that anger may lead individuals to blame others for negative events and believe he or she can still have positively influence the situation (Lerner and Tiedens, 2006). This is particularly important in escalation situations because the decision maker may experience anger when he or she receives information about the project’s lack of success. Accordingly, this anger may lead the decision maker to blame others for the negative events while still hoping that the decision maker could turn the project around. Indeed, one reason people continue escalating their commitment to a failing course of action is because they think they can turn the failing project around (Sleesman et al., 2012).

The story for confidence is slightly different. Our results demonstrated that the lagged effect of confidence *did* influence one’s future decision to escalate. However, the lagged effect of confidence did not influence the proportion of funds invested in one’s future decision. Therefore, it appears that those who had higher confidence were more likely to continue escalating but were no more likely to invest more money into the project. This is somewhat similar to the findings of Ronay et al. (2017) who demonstrated that overconfidence predicts escalation behavior in public but not private settings. Thus, the reciprocal relationship between escalation of commitment and confidence is one that is less straightforward than the reciprocal relationship between escalation of commitment and anger.

### Implications

These results have important implications for practitioners. At a basic level, this study provides good and bad news regarding escalation of commitment over time. First, this study shows that while not everyone continually persists with a failing course of action, a substantial proportion of individuals in the current study (68%) never abandoned the project. Though this finding is noteworthy, one must recognize that the 68 percent may be an overestimation, as participants were not investing their own money and may have felt they could make riskier decisions. For instance, people tend to make riskier financial decisions when the money used is not their own (Chakravarty et al., 2011). However, while this value may be an overestimation, it is nevertheless noteworthy especially when considering the notion that a manager making investment decisions for a company in many circumstances is not using his or her own money.

### Limitations and Future Directions

One of the primary limitations of this study is the sample we used. Notably, our sample consisted of undergraduate students completing an artificial task. Therefore, our sample may not be representative of real organizational decision makers. It is hard to say students, who are not investing their own money and from whom there are no true consequences to escalating, are comparable to a manager who is investing company resources with their job on the line. It is plausible that a manager would exhibit higher concern for the failure of the project. However, the focus of this study was on basic decision-making processes and the psychological outcomes of escalating. Had the consequences of decisions been real (i.e., actual loss of money and resources) the results may have differed. Nevertheless, we took steps to increase the fidelity of the situation, and participants appeared to take the task seriously (i.e., not arbitrarily quitting or continuing with the study). Future research should replicate the results of the study using a sample of managerial decision makers. An additional limitation of this study is that we only examined one continuously failing project. In reality, projects may have periods of success in addition to setbacks. Future research should investigate how participants would react if they are given a glimmer of hope for the project to recover, only to have it ultimately fail in the end.

An additional goal for future researchers is to examine the individual differences, such as guilt, shame, or pride, that lead some people to continually escalate and others to abandon a failing course of action. The results of such research could assist organizations in selecting individuals who are less likely to waste organizational resources. For example, future researchers should examine the role of the dark triad (narcissism, psychopathy, and Machiavellianism; Paulhus and Williams, 2002) on escalation of commitment over time. Given the destructive nature of these traits and their relations with job performance and counterproductive work behaviors (O’Boyle et al., 2012), one would expect that individuals high on these traits would not feel guilty about wasting an organization’s resources. Therefore, a positive relationship may be expected between the dark triad and escalation of commitment over time. In addition, future research should seek to try to increase the ecological validity in decision making tasks in order to decrease the chance participants are taking risks simply because there are no consequences.

Our results also demonstrate that whereas people do escalate, they become less confident in their decision and ultimately invest decreasing proportions of available funds over time. This suggests that individuals may actually recognize that they are making irrational decisions by escalating. In accordance with self-justification theory, individuals may justify their escalation actions by investing fewer and fewer resources over time. The good news for organizations is that while people may tend to make irrational decisions, they waste proportionally fewer organizational resources over time. That said, decision makers should be afforded the opportunity to abandon failing endeavors, without the possibility of negative consequences, as people may actually recognize the irrationality of the decision to escalate and may be more inclined to abandon a failing course of action if there are not possible negatives consequences associated with abandoning.

## Conclusion

Our study contributes to the escalation literature in two meaningful ways. First, we demonstrated that escalation of commitment occurs over time, and that over half of the participants escalated through all five decisions. Second, we examined the impact of escalating on psychological consequences, such as anger and confidence. We found that with each decision individuals become angrier and less confident with their decisions. Additionally, the choice to escalate negatively impacts confidence, which then positively predicts the choice to escalate in the next decision.

Evidence of the effects found in the current study is present in well-known examples of escalation of commitment, such as Tesco’s withdrawal from United States markets. After nearly 6 years of continued efforts, Tesco, one of Britain’s largest supermarket companies, eventually chose to abandon their failing attempt to enter the United States supermarket industry (Werdigier, 2013). Some estimates of the cost for Tesco to withdraw from United States markets were as high as £1.5 billion on top of the $22 million each month the company was losing on its Fresh and Easy supermarket chain (Butler, 2013). In March 2008, Tesco began slowing its expansion of stores in the United States and the long-term viability of the project was increasingly in question (Best, 2012). Thus, confidence waned over time and investments became increasingly smaller. In the case of Tesco, those tasked with making optimal decisions often irrationally escalated their commitment, and such irrational decision making clearly came at a great cost.

## Ethics Statement

Rick Scheidt, Kansas State University IRB Committee Chair (785) 532-1483 rscheidt@ksu.edu. Prior to participating in this research, participants provided written informed consent. Participants also had an opportunity to ask any questions regarding the study prior to signing the informed consent. After providing written informed consent, participants were presented with the decision-making tasks in this research.

## Author Contributions

AJ, SH, and EK conceptualization. AJ and SH data collection. AJ and MY data analysis. AJ, SH, EK, MY, and ML manuscript preparation and review.

## Conflict of Interest Statement

The authors declare that the research was conducted in the absence of any commercial or financial relationships that could be construed as a potential conflict of interest.

## Footnotes

1 We would like to thank a reviewer for highlighting this point.

2 The nature of the task does not allow the time slope to vary across subjects because it produces an identical slope for each participant. When a participant chooses to abandon the task, the value for escalation becomes 0. Accordingly, we did not allow the slopes to vary for participants.

## APPENDIX A

### Escalation of Commitment Vignettes

#### Decision 1

You are the Vice President of Operations for a mid-sized high-tech manufacturing firm. You have 10 million dollars and 3 years to complete a research project that will develop a radar-scrambling device that would render a ship undetectable by conventional radar, in effect, a radar-blank ship. Prior to the beginning of the project, Steve, the project engineer, informs you that he does not think that all 10 million dollars will be needed to successfully complete the project, but he does think that he will need at least 5 million dollars to complete the project.

Between 5 million dollars and 10 million dollars, how much money would you like to invest in the project? $________________

#### Decision 2

Two years after the project started, Steve retired from the company. Jackie is the new project engineer. You meet with Jackie to get an update on the project. Jackie informs you that Steve used the money you initially invested to purchase inexpensive materials that are of poor quality. As a result, all of the computer components in the plane keep short-circuiting. Jackie says that she is certain she can remedy the mistake, but that she will need an additional 3 million to 6 million dollars in funding. The decision you face now is to either abandon the project or authorize more funding to continue this radar-scrambling project.

Authorize more funding _______

Abandon the project _______

Between 3 million dollars and 6 million dollars, how much money would you like to authorize to continue the radar-scrambling plane? $________________

#### Decision 3

Three months after you provided the additional funding for Jackie to replace the faulty parts that Steve had purchase, you ask Jackie for an update on the project. You are pleased to learn that the computer components are now working properly. Jackie also informs you that she believes the project will be finished on schedule. However, she informs you that the radar-scrambling device also scrambles other electronic devices, such as the pilot’s communication system. She informs you that the problem can be fixed with a new software system for the radar-scrambler, but that she needs an additional 2 million to 5 million funds to purchase the new software system. The decision you face now is to either abandon the project or authorize more funding to continue this radar-scrambling project.

Authorize more funding _______

Abandon the project _______

Between 2 million dollars and 5 million dollars, how much money would you like to authorize to continue the radar-scrambling plane? $________________

#### Decision 4

After another 3 months have passed, you visit the engineering department to view the radar-scrambling plane. You are pleased to learn that the additional funding you granted solved the problem with the radar-scrambler affecting other devices. Jackie informs you that the plane is ready for a test flight. She asks if you would like to ride aboard the plane during the test flight. You are excited to see how well the plane is working and decide to ride aboard the plane. During the test flight everything works perfectly. None of the radar systems are detecting the plane. 30 min after take-off, the pilot informs you the test is over and he is landing the plane. Once on the ground, you ask the pilot why he landed the plane so shortly after the flight began. He informs you that the additional weight of the radar-scrambling device caused the plane to burn the fuel faster than expected. The pilot suggests that the fuel tanks be upgraded to allow for longer flights but that it would cost an additional 4 million to 7 million dollars in funding. The decision you face now is to either abandon the project or authorize more funding to continue this radar-scrambling project.

Authorize more funding _______

Abandon the project _______

Between 4 million dollars and 7 million dollars, how much money would you like to authorize to continue the radar-scrambling plane? $________________

#### Decision 5

Three months later, you discover that another firm has already begun marketing a similar product that takes up less space and is much easier to operate than your design. Jackie informs you that the project is 90% complete. She informs you that she is pleased with all of the progress that has been made despite the issues that have arisen along the way. Jackie informs you that although the upgraded fuel tanks allow the plane to fly much further than before, the fuel tanks cost more than expected. She informs you that she will need an additional 1 million to 4 million dollars in funding to pay for the remainder of the project. The decision you face now is to either abandon the project or authorize more funding to continue this radar-scrambling project.

Authorize more funding _______

Abandon the project _______

Between 1 million dollars and 4 million dollars, how much money would you like to authorize to continue the radar-scrambling plane? $________________
